# A Method to Train Marmosets in Visual Working Memory Task and Their Performance

**DOI:** 10.3389/fnbeh.2018.00046

**Published:** 2018-03-16

**Authors:** Katsuki Nakamura, Reiko Koba, Miki Miwa, Chieko Yamaguchi, Hiromi Suzuki, Atsushi Takemoto

**Affiliations:** Cognitive Neuroscience Section, Primate Research Institute, Kyoto University, Inuyama, Japan

**Keywords:** visual working memory, non-human primate, marmoset, delayed matching-to-sample task, neurodegenerative diseases, aging

## Abstract

Learning and memory processes are similarly organized in humans and monkeys; therefore, monkeys can be ideal models for analyzing human aging processes and neurodegenerative diseases such as Alzheimer’s disease. With the development of novel gene modification methods, common marmosets (*Callithrix jacchus*) have been suggested as an animal model for neurodegenerative diseases. Furthermore, the common marmoset’s lifespan is relatively short, which makes it a practical animal model for aging. Working memory deficits are a prominent symptom of both dementia and aging, but no data are currently available for visual working memory in common marmosets. The delayed matching-to-sample task is a powerful tool for evaluating visual working memory in humans and monkeys; therefore, we developed a novel procedure for training common marmosets in such a task. Using visual discrimination and reversal tasks to direct the marmosets’ attention to the physical properties of visual stimuli, we successfully trained 11 out of 13 marmosets in the initial stage of the delayed matching-to-sample task and provided the first available data on visual working memory in common marmosets. We found that the marmosets required many trials to initially learn the task (median: 1316 trials), but once the task was learned, the animals needed fewer trials to learn the task with novel stimuli (476 trials or fewer, with the exception of one marmoset). The marmosets could retain visual information for up to 16 s. Our novel training procedure could enable us to use the common marmoset as a useful non-human primate model for studying visual working memory deficits in neurodegenerative diseases and aging.

## Introduction

Learning and memory processes are similarly organized in humans and monkeys (Zola-Morgan and Squire, 1985, 1993; Squire, 1987). Behavioral and neuropsychological studies in monkeys can therefore elucidate human memory processes and associated brain structures, neurodegenerative diseases involving dementia (e.g., Alzheimer’s disease), and aging processes. As impairment of visual working memory is a prominent symptom of neurodegenerative diseases and aging (Oscar-Berman and Bonner, 1985; Sahakian et al., 1988; Sahgal et al., 1992; Grady et al., 1998), the use of appropriate methods for evaluating visual working memory is crucial. The delayed matching-to-sample task is a powerful tool for such evaluation in humans and monkeys (Squire, 1992; Paule et al., 1998), and performance on this task can predict Alzheimer’s disease in patients with mild cognitive impairment (Didic et al., 2013). Performance on such tasks is known to depend on temporal structures (Fuster et al., 1981; Horel et al., 1987; Cirillo et al., 1989; Gaffan and Murray, 1992) as well as frontal cortices (Passingham, 1975; Bauer and Fuster, 1976).

With the emergence of novel gene editing technologies, common marmosets (*Callithrix jacchus*) have been suggested as a promising non-human primate model for neurodegenerative diseases (‘t Hart et al., 2012; Kishi et al., 2014). The common marmoset’s lifespan is approximately 15 years (Smucny et al., 2004; Ross et al., 2012), which is relatively short for non-human primates. Therefore, the common marmoset may be a practical non-human primate model for human aging processes (Fischer and Austad, 2011; Tardif et al., 2011; Munger et al., 2017). Common marmosets are small New World monkeys of the Callitrichidae family (Ryland, 1993; Fischer and Austad, 2011) and frequently exhibit species-specific social behavior, such as cooperative breeding, food sharing, and vocal communication (Ryland, 1993). Marmosets are endemic to the tropical rainforests of eastern Brazil and are believed to rely on spatial memory for foraging, which has led to much research into their spatial memory (MacDonald et al., 1994; Easton et al., 2003; Spinelli et al., 2004; Yamazaki et al., 2016).

However, no data are available for their visual working memory. Several studies have reported difficulties in training marmosets to perform a delayed matching-to-sample task (Spinelli et al., 2004) and an easier version called a delayed non-matching-to-sample task (Ridley et al., 1988). This is likely due to the lack of tasks designed specifically for marmosets and lack of training procedures (Mitchell and Leopold, 2015). Here, we developed a novel procedure for training marmosets in a delayed matching-to-sample task in combination with training in visual discrimination and reversal tasks (Takemoto et al., 2011, 2015).

## Materials and Methods

### Animals

A total of 13 young common marmosets (10 males and 3 females, aged 1 year and 7 months to 4 years and 1 month; **Table 1**) were used in this study. The marmosets were reared in rooms at temperatures of 26–30°C and relative humidities of 25–60% at the Primate Research Institute, Kyoto University, and were maintained on a standard 12:12-h light/dark cycle with lights turned on at 07:00. They received 25 g of New World Monkey pellets (SPS, Oriental Yeast Co., Ltd., Tokyo, Japan) and additional food supplements such as bananas, apples, raisins, and mealworms twice daily with *ad libitum* access to water. All marmosets were initially naïve to cognitive testing, including the delayed matching-to-sample task. This experiment was approved by the Kyoto University Animal Care and Use Committee and conducted in accordance with Japanese law and the Guide for Care and Use of Laboratory Primates of the Primate Research Institute, Kyoto University.

**Table 1:** Characteristics and training history of subject marmosets used in this study.

Subject	Sex	Age	I	II(SS)	II(VD)	III	IV	V	Delay	Gen
S1	M	4 years 1 month	✓	✓		✓	✓	✓	✓	✓
S2	M	3 years 1 month	✓	✓		✓	✓	✓	✓	✓
S3	M	2 years 1 month	✓		✓	✓	✓	✓	✓	✓
S4	M	1 year 9 months	✓		✓	✓	✓	✓	✓	✓
S5	M	2 years 1 month	✓		✓	✓	✓	✓	✓	✓
S6	M	1 year 11 months	✓		✓	✓	✓	✓	✓	
S7	F	1 year 10 months	✓		✓	✓	✓			
S8	F	1 year 7 months	✓	✓	✓	✓	NA			
S9	F	1 year 10 months	✓		✓	✓				
S10	M	1 year 10 months	✓		✓	✓				
S11	M	1 year 11 months	✓		✓	✓				
S12	M	1 year 8 months	✓	✓	✓	NA				
S13	M	1 year 10 months	✓	✓	✓	NA				

*Delay, delay length variation; F, female; Gen, generalization to stimuli; M, male; NA, not achieved; SS, small step training; VD, visual discrimination training*.

### Behavioral Paradigms and Training Procedures

The experiment was conducted for 5 days in a week (usually from Monday to Friday) between 11:00 and 15:00 in a colony housing approximately 30 marmosets. During the experiment, the subject marmoset retained visual, auditory, and olfactory contact with other marmosets in the same colony.

**FIGURE 1:**
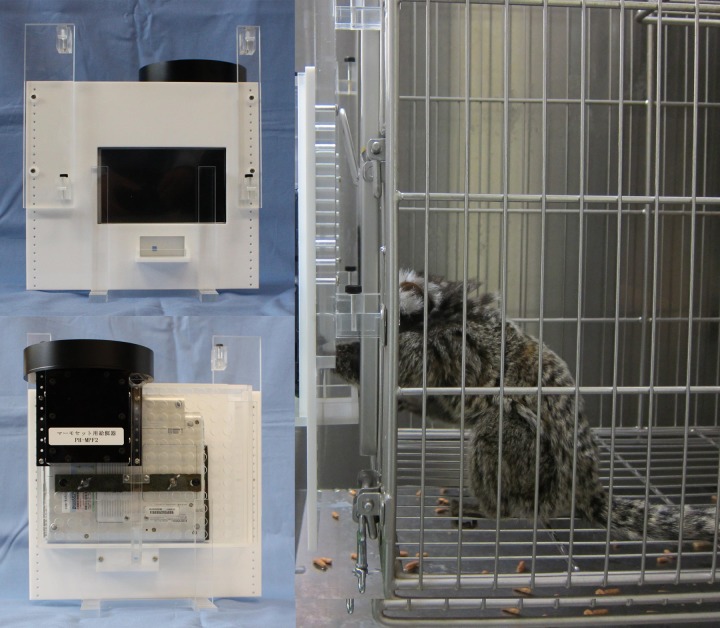
Apparatus. Front view of the apparatus (top left). The apparatus was 30 cm (W) × 20 cm (D) × 25 cm (H) in size, and the screen was 154 mm (W) and 91 mm (H) in size (1024 pixels × 600 pixels). A food tray was located underneath the screen. Back view of the apparatus (bottom left). A black USB-powered feeder was attached to the acrylic panel. A marmoset touching the screen (right). The marmoset typically crouched in the testing cage to touch the screen or pick up a reward. The distance between the screen of the apparatus and the front panel of the cage was approximately 45 mm.

An apparatus developed for marmosets was used (Takemoto et al., 2011). Briefly, the apparatus consisted of three components: (1) a mini laptop PC with a touch-sensitive screen (Model SC, Kohjinsha, Tokyo, Japan), (2) a USB-powered feeder, and (3) an acrylic case (**Figure 1**, left panel). The size of the apparatus was 30 cm (W) × 20 cm (D) × 25 cm (H), and the screen was 154 mm wide and 91 mm tall (1024 pixels × 600 pixels). The mini laptop PC controlled and stored all events including the stimulus presentation and touch-response acquisition with custom software using Microsoft Direct X technology, and supplied electric power to the feeder.

The apparatus was attached to the front panel of the cage by the four hooks of the acrylic case in order that the touch-sensitive screen was exposed to the marmoset. Underneath the screen, the case had a food tray to deliver a reward. The distance between the screen and the front panel was 45 mm, and the marmoset could touch the screen and pick up the reward from the tray. As illustrated in the right panel of **Figure 1**, the marmoset crouched in the cage while touching the screen or retrieving a reward. The apparatus was attached to the cage only 30 s before the experiment, and was removed after the experiment. After a daily session, the apparatus was cleaned with a wet dust cloth to minimize the effect of scents on performance. In most experiments, the same apparatus was used for the same marmoset.

During the experiments, the experimenter was in a small curtained space and video-monitored and -recorded the marmoset behavior via network cameras (VB-C60B, Cannon, Tokyo, Japan). Only when the experimenter reset the rewards in the apparatus and the software in the PC could the subject marmoset see the experimenter.

Solid food rewards of approximately 3 mm diameter were made by the researchers and technicians. The rewards were made from 12 main ingredients (gum powder, soybean powder, marshmallow, sponge cake, egg cookie, nuts, sweet potato, cheese etc.). To maintain the motivation of each marmoset, four different rewards were typically selected for each daily session based on the preference of the marmoset. Food and water restriction was not applied in these experiments.

The training occurred in five phases. Shaping, which was introduced by Skinner (1938), is a conditioning method for animals, in which a complex task is decomposed into several sub-tasks, thereby providing an easier method for learning the complex task (Krueger and Dayan, 2009). Such small-step task training (so-called “successive approximation”) was adopted for the marmosets in this study.

**FIGURE 2:**
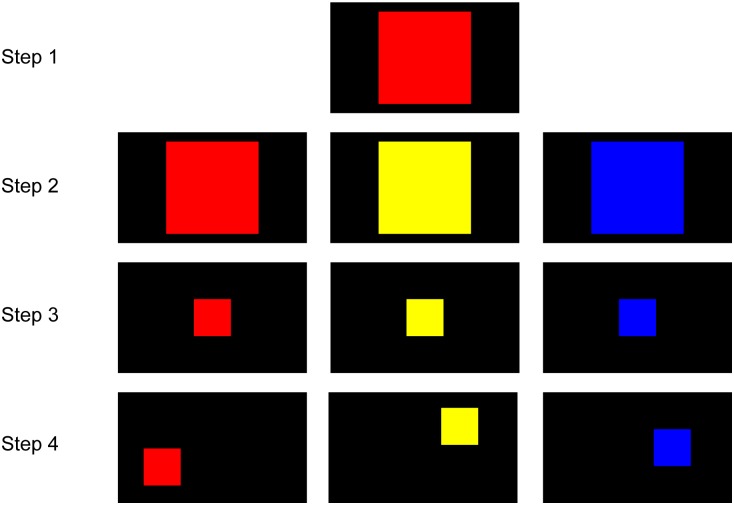
Steps for touch training. In Step 1, the marmosets touched any part of a large red square (500 pixels × 500 pixels) on the screen to receive a reward. In Step 2, the color of the square was randomly set to red, yellow, or blue. In Step 3, the size of the square gradually decreased to the final size (200 pixels × 200 pixels). In Step 4, the location of the square varied from trial to trial.

In the first phase, the marmosets were trained to touch a colored square on the screen to obtain a reward (**Figure 2**). In Step 1, the marmosets initially received a reward for touching any point within a large red square (500 pixels × 500 pixels) at the screen center. In Step 2, the color of the square was randomly set to red, yellow, or blue in different trials. In Step 3, the size of the square was gradually decreased to the final size (200 pixels × 200 pixels) that was used for all stimuli in further training. In Step 4, the location of the square on the screen varied in different trials. The marmoset was considered to have completed this phase when it could rapidly touch the target square in every trial. If the marmoset rarely touched the screen, food was presented in front of the screen to attract the marmoset to the screen. It was not necessary for the marmosets to be familiarized to the apparatus, most likely because young marmosets are very curious.

**FIGURE 3:**
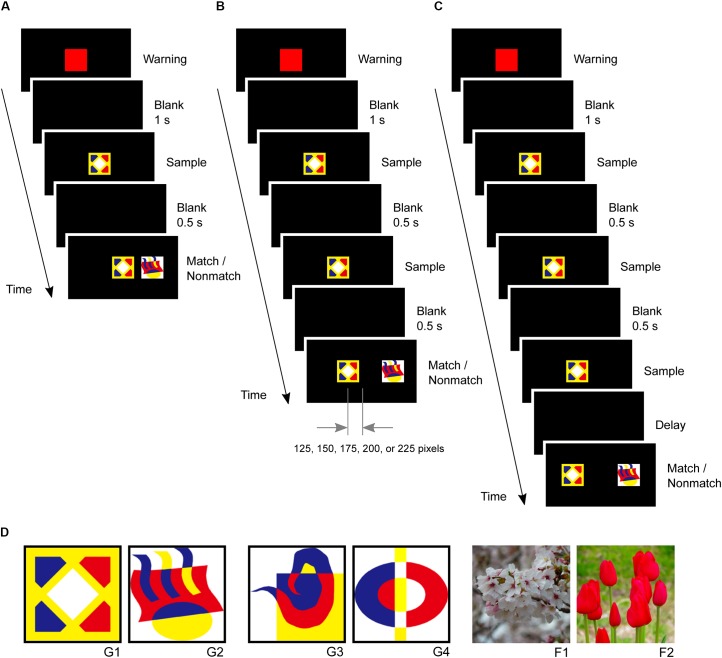
Delayed matching-to-sample task and visual stimuli. **(A)** After the marmosets touched the sample stimulus, a matching stimulus was presented at the same center position, and a non-matching stimulus was presented 250 pixels right or left of the center. **(B)** A sample stimulus was presented twice to ensure that the marmosets looked at it. The non-matching stimulus was always located 250 pixels right or left of the center, but the position of matching stimulus gradually shifted 0, 125, 150, 175, 200, 225, and 250 pixels from the center. **(C)** Task events in a trial of the delayed matching-to-sample task. The same sample stimulus was presented thrice. **(D)** Visual stimuli used in this study, including four graphic patterns (G1, G2, G3, and G4) and two flower photographs (F1 and F2).

In the second phase, small-step task training was introduced to five marmosets since previous studies have reported difficulties in training marmosets to perform a delayed matching-to-sample task (Spinelli et al., 2004). The marmosets were first trained to touch a red square warning stimulus followed by a graphic pattern sample stimulus at the center of the screen. After the marmosets touched the sample stimulus, a matching stimulus appeared at the same center position while a non-matching stimulus appeared 250 pixels right or left of the center. The marmosets received rewards for touching the matching stimulus (**Figure 3A**). In the second step, the sample stimulus was presented twice after the warning stimulus to ensure that the marmoset looked at it. After the second touching of the sample stimulus, the matching and non-matching stimuli were presented simultaneously. The non-matching stimulus was always located 250 pixels right or left of the center, while the matching stimulus gradually shifted 0, 125, 150, 175, 200, 225, and 250 pixels from the center as this step progressed. The marmosets received a reward for touching the matching stimulus. Four touches were required in this step (**Figure 3B**). The marmosets typically spent 2 days completing these steps.

This small-step training was successful in three out of the five marmosets. The remaining two marmosets failed to learn the task. To direct the marmosets’ attention to the visual stimuli and improve their touching, 11 marmosets including the two failed marmosets were trained in visual discrimination and reversal tasks rather than the small-step task training in the second phase. The tasks and stimuli were the same as those described previously (Takemoto et al., 2015). In the visual discrimination task, a red square was presented at the center of the screen as a warning signal until the marmoset touched it. A pair of graphic patterns were then presented simultaneously at positions 250 pixels left and right of the center of the screen. The left–right positions were pseudo-randomly changed and counter-balanced. One of the patterns was always associated with a reward and the other was not. When the marmoset correctly touched the reward-associated pattern, a reward was delivered. The correct and incorrect responses were followed by 3 and 5 s inter-trial intervals, respectively. After achieving 90% correct response rates (≥90 correct responses in 100 trials), the marmoset progressed to the next visual discrimination learning problem using another pair of stimuli. After the third visual discrimination learning problem, reversal learning was introduced the following day. In reversal learning, the stimulus–reward association was opposite to that in the preceding visual discrimination learning problem. Reversal learning was repeated at least twice for each marmoset.

In the third phase, the marmosets were trained in the delayed matching-to-sample task, which required them to touch a warning stimulus and a sample stimulus three times. The sample stimulus was presented three times to ensure that the marmosets looked at it and paid attention to it. The matching and non-matching stimuli were then presented simultaneously at positions 250 pixels left or right of the center (**Figure 3C**). The marmosets received rewards for touching the matching stimulus. Both correct and incorrect trials were followed by a 3 s inter-trial interval. A single session consisted of 28 trials. The marmosets were considered to have completed this phase if they achieved 80% correct response rates (≥23 correct responses in 28 trials) in five consecutive sessions. Only one stimulus pair (G1 and G2 in **Figure 3D**) was used in this phase.

The final size of the stimuli (200 pixels × 200 pixels) was based on the size of the screen and the proficiency of the marmosets’ touching response. If the marmoset looked at the stimulus when positioned 80 mm from the screen, this stimulus size corresponded to 21° (W) × 21° (H) and the screen covered 88° × 59° in visual angle. The light condition in the cage was between 40 and 330 LUX when the apparatus was attached, depending on the location of the cage in the colony. Four colors were used to create visual stimuli. The luminance and chromaticity coordinate (CIE x, y) of blue, yellow, red, and white were 6.4 cd/m^2^ and (0.16, 0.07), 140.4 cd/m^2^ and (0.46, 0.44), 25.8 cd/m^2^ and (0.63, 0.31), and 162.7 cd/m^2^ and (0.32, and 0.27), respectively. The luminance of background was 0.5 cd/m^2^.

In the fourth phase, a new pair of visual pattern stimuli were introduced (G3 and G4 in **Figure 3D**), with each session using the new pair in 24 trials and the previous pair in four trials. The marmosets completed this phase by achieving a >80% correct response rates in five consecutive sessions. In the fifth phase, the 4 graphic pattern stimuli were intermingled such that two were randomly selected for each trial. The completion criterion was the same as that in the third phase.

Typically, the subject marmoset repeated 120 trials of the visual delayed matching-to-sample task in a session. The length of a daily session was typically 1 h (up to 1.5 h). Twenty-four rewards could be set in the feeder at any one time; therefore, 24 trials were set in a block. After 24 trials were completed, the program was reset to continue the session and rewards were added to the feeder. A resting time was never set during a daily session, but it took 3–5 min to reset the program and rewards. If the marmoset did not touch the screen for 15 min, the session was aborted. If a marmoset failed to meet the completion criterion within 5000 trials in the third or fourth phase, it was regarded as unable to complete the phase and training was aborted. All inter-stimulus intervals were 0.5 s long.

### Delay Length Variation

Longer sample-comparison delays were introduced to evaluate visual working memory in the six marmosets that completed the fifth phase. Three delay lengths were used in each session; 0.5, 2.0, and 4.0 s in the short delay condition; 0.5, 4.0, and 8.0 s in the medium delay condition; and 0.5, 8.0, and 16.0 s in the long delay condition. With each session featuring three delay lengths, 12 match/non-match combinations (from four stimuli), and two correct locations (left or right), there were 72 trial types, which were presented 5 times each for 360 trials per delay condition. Within-subject ANOVA was used to compare the response accuracies in the 0.5 s delay trials across different conditions, and paired *t*-tests were used to compare the response accuracies in the 4.0 s delay trials across the short and middle delay conditions and in the 8.0 s delay trials across the middle and long delay conditions. The performances of the marmosets were evaluated to determine whether each marmoset achieved above-chance performance in the 16.0 s delay trials using a test for proportion.

To directly compare the marmosets’ performance levels with those of other animals, “zero-delay performance” and “performance half-life,” as defined by Lind et al. (2015), were calculated. Zero-delay performance is the performance when matching and non-matching stimuli are presented immediately after the sample stimulus, and reflects the performance required for minimum visual working memory requirements. A performance half-life is the delay length at which the performance equals half the difference between the zero-delay performance and the chance performance.

### Generalization to Novel Stimuli

Finally, the learning abilities of five marmosets in response to novel visual stimuli using two photographs of flowers (F1 and F2 in **Figure 3D**) that were unfamiliar to the marmosets. The number of trials required to obtain a >80% correct response rate (≥23 correct responses in 28 trials) was evaluated in five consecutive trials. Paired *t*-tests were then used to compare the number of trials needed for graphic pattern stimuli (as in the third phase) and the number needed for the photographs.

## Results

### Learning the Matching-to-Sample Task

**FIGURE 4:**
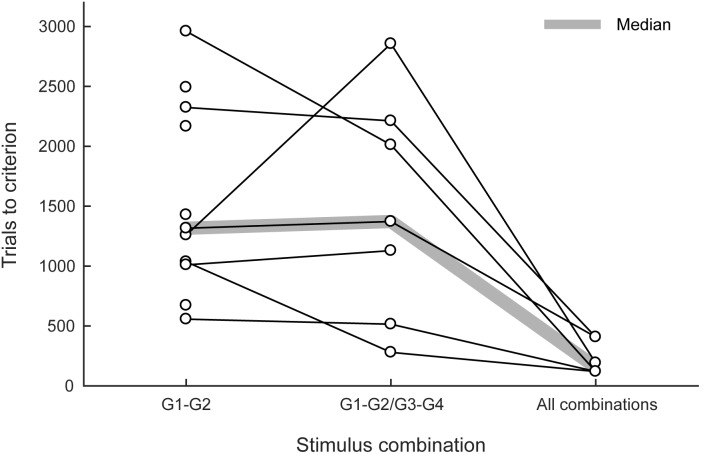
Trials needed to meet the proficiency criteria in the delayed matching-to-sample task. The numbers of trials needed to meet the proficiency criteria in the third (left), fourth (center), and fifth phases (right) are summarized. G1, G2, G3, and G4 represent the graphic patterns used as stimuli. The thick shaded line indicates median values.

All 13 marmosets (100%) completed the first training phase within 22 days. Five marmosets were then trained in the small-step task training (the second phase). Three marmosets cleared the second phase but two failed. The two marmosets that failed exhibited strong position preference when the distance of the matching stimulus from the center increased to 225 pixels. Eleven new marmosets (including the two marmosets that failed) were trained in the visual discrimination and reversal tasks for 9–19 days. All 13 marmosets were trained in the third phase; 11 of them (85%) met the proficiency criterion for the delayed matching-to-sample task, and 557–2960 trials (median: 1316 trials) were found to be necessary (left column in **Figure 4**). All of the marmosets trained in the visual discrimination and reversal tasks, but not in the small-step task, met the criterion, with the exception of the two marmosets that failed the second phase. Again, these two marmosets exhibited strong position preference but not pattern preference.

**FIGURE 5:**
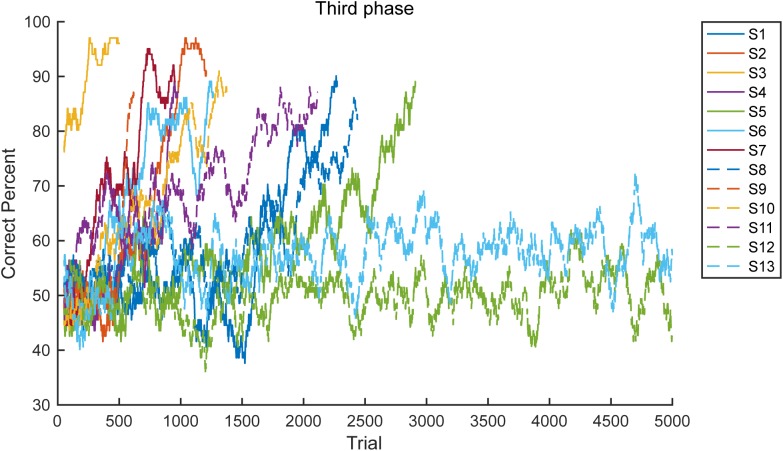
Learning curve. Learning curves of all 13 subjects (S1–S13) in the third phase are presented. Two subjects failed to meet the proficiency criteria in 5000 trials.

**Figure 5** provides the learning curves for all 13 marmosets in the third phase. The graph illustrates the simple moving average from 101 trails. As clearly demonstrated, the marmosets met the criterion soon after achieving a >70% correct response rate (*n* = 11, median 617 trials). However, the two marmosets that failed did not achieve a >70% correct response rate.

Eight of these 11 marmosets were advanced to the fourth phase; seven of them (88%) met the proficiency criterion, and 280–2856 trials (median 1372 trials) were found to be necessary (center column in **Figure 4**). The one marmoset that failed was initially trained with the small-step method in the second phase. Six of these seven marmosets were advanced to the fifth phase; all of them (100%) rapidly met the proficiency criterion, and 120–552 trials (median: 156 trials) were found to be necessary (right column in **Figure 4**).

### Effect of Delay Length

**FIGURE 6:**
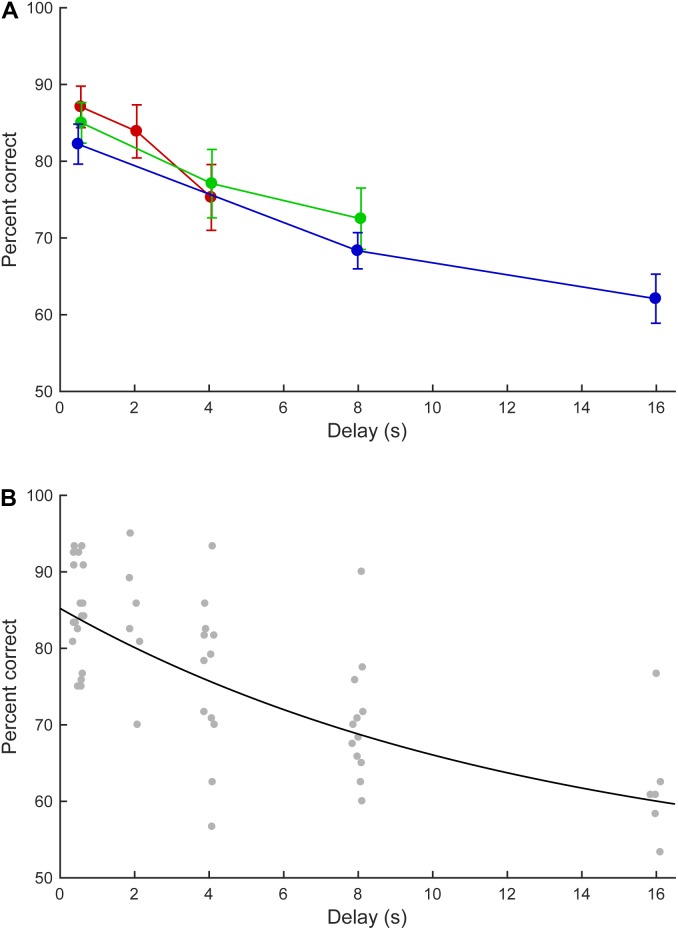
Delayed matching-to-sample task performance as a function of delay length. **(A)** Mean performance of the marmosets in the short (red line), middle (green), and long (blue) delay conditions. The error bars represent standard errors. **(B)** Estimated performance curve based on the parameters described by Lind et al. (2015). The data for individual marmosets are indicated by gray dots.

For the six marmosets that completed the fifth phase, the effect of delay length on task performance was examined. **Figure 6A** summarizes the marmosets’ performance in the short, medium, and long delay conditions. In the short delay condition, the correct response rates were 75.8–93.3% (median: 88.3%), 70.0–95.0% (median: 84.2%), and 56.7–85.8% (median 78.8%) for the 0.5, 2.0, and 4.0 s delays, respectively. In the medium delay condition, the correct response rates were 75.0–93.3% (median: 83.8%), 62.5–93.3% (median: 76.7%), and 62.5–90.0% (median: 70.8%) for the 0.5, 4.0, and 8.0 s delays, respectively. In the long delay condition, the correct response rates were 75.0–92.5% (median: 81.7%), 60.0–77.5% (median: 67.9%), and 53.3–76.7% (median: 60.8%) for the 0.5, 8.0, and 16.0 s delays, respectively. In all delay conditions, longer delays were associated with lower correct response rates. The performances at equal delays (0.5, 4.0, or 8.0 s) did not significantly differ across conditions (0.5 s delay: within-subject ANOVA, *F*(2, 10) = 2.197, *P* = 0.162; 4.0 s delay: paired *t*-test, *t*(5) = -0.781, *P* = 0.47; 8.0 s delay, paired *t*-test, *t*(5) = 1.111, *P* = 0.317).

With the 16.0 s delay, five out of six marmosets achieved correct response rates of >58.3% (60.8, 62.5, 76.7, 60.8, and 58.3%), corresponding to ≥70 correct responses in 120 trials. These five marmosets exhibited significantly above-chance performances (test for proportion, *P* < 0.05, *Z* = 2.37, 2.74, 5.85, 2.37, and 1.82, respectively). The remaining marmoset achieved a correct response rate of 53.3% that did not significantly differ from chance performance (*P* = 0.26, *Z* = 0.72). These data suggest that most marmosets could retain visual information for 16.0 s.

The zero-delay performance of the marmosets was 85.2% and the performance half-life was 8.83 s. **Figure 6B** provides the marmosets’ estimated performances.

### Generalization to Novel Stimuli

Four of the five marmosets (80%) rapidly met the proficiency criterion when presented with the novel flower stimuli, and 364–476 trials (median: 406 trials) were found to be necessary. For the marmoset that did not achieve proficiency in 1500 trials, a new pair of flower photographs were presented, and the proficiency criterion was then met in only 140 trials. The marmoset may have disliked the first pair of photographs for some reason. These five marmosets required significantly fewer trials to achieve proficiency in the flower photographs task than they did in the initial matching-to-sample training of the third phase (paired *t*-test, *P* < 0.0001). These data suggest that once the marmosets had learned the rules of the task, they could quickly adapt to novel stimuli.

## Discussion

We achieved our goal of developing an effective procedure for training marmosets in a visual delayed matching-to-sample task, thereby providing the first evidence that common marmosets can learn the task. After training the marmosets to touch colored squares, we trained them in visual discrimination and reversal tasks, which we believe was a very important step in directing their attention to details of the visual stimulus, including shape, color, etc., Attending to such details of the visual stimuli likely helped the marmosets to memorize the visual pattern and conduct same-different discrimination. We initially adopted shaping introduced by Skinner (1938). We decomposed the delayed matching-to-sample task, and trained the marmosets in a small-step manner (so-called “successive approximation”). Training of visual discrimination and reversal tasks was highly successful. All new eight marmosets learned the visual matching-to-sample task. However, the two marmosets that failed the second phase also failed to learn the visual matching-to-sample task. Our small-step task training allowed the marmosets to touch the same central position repeatedly to get a reward. Therefore, the two marmosets that failed might have attended only to the position but not to the details of visual stimulus patterns. Indeed, both marmosets exhibited strong position preference. Even after we trained these two marmosets in the visual discrimination and reversal tasks, the position preference in the delayed matching-to-sample task could not be corrected. The sequence of task events was very different between the delayed matching-to-sample and visual discrimination tasks, and the marmosets could recognize which task they were performing. Once bad habits were learned in a task, it was very difficult to correct these habits when the task was repeated. This may explain why the two marmosets that failed were unable to learn the delayed matching-to-sample task after they learned the visual discrimination and reversal tasks. The marmoset that failed to meet the criterion in the fourth phase was also trained in the small-step manner. We then concluded that the small-step task training is not necessary, and may cause problems when training marmosets in the delayed matching-to-sample task.

With this method, we successfully trained 85% of the marmosets in the visual delayed matching-to-sample task. The marmosets initially required many trials to learn the task (median: approximately 1300 trials), but once they achieved a >70% correct response rate, they learned the task relatively soon after. Furthermore, once they learned the task rules, they could rapidly generalize them to novel photographic stimuli (median: 406 trials). In models of neurodegenerative diseases or aging, the marmosets’ performance should gradually decline. Our present results suggest that once the marmosets learned the task rule in advance, we could evaluate and trace their disease- or age-related cognitive decline.

To examine the marmosets’ ability to retain visual information, we tested their working memory with delays of up to 16.0 s. All but one marmoset achieved significantly above-chance performance even at the 16.0 s delay. The marmosets’ zero-delay performance and performance half-life were 85.2% and 8.83 s, respectively. These results confirmed that the marmosets could retain the visual information for up to 16.0 s. In addition, these values were very similar to those of other non-human primates, such as pig-tailed and squirrel monkeys (Lind et al., 2015). Thus, the marmoset could be a good non-human primate model for studying memory processes.

The marmosets achieved task proficiency without any food or water deprivation. Furthermore, we conducted this experiment in their colony where they had visual, auditory, and olfactory contact with each other. Other marmosets occasionally made noises and vocalizations, which may have disturbed the subject marmosets. Therefore, our results may underestimate the marmosets’ potential performance. Nevertheless, the marmosets exhibited adequate performance levels for surveying their visual working memory. By using a compact apparatus developed for marmosets (Takemoto et al., 2011) in the colony, we could train and test several marmosets simultaneously. This saved time and space and thus enabled efficient testing. This is particularly important for projects comparing the behaviors of disease model and control animals, as it allows for the assessment of several individuals per group.

In summary, our findings suggest that the common marmoset could be an important model for studying memory deficits in neurodegenerative diseases and aging.

## Author Contributions

KN planned this project and acquired the funding. KN, RK, and AT designed the study. RK, MM, CY, HS, and AT collected the experimental data. KN and AT analyzed the experimental data and prepared the manuscript.

## Conflict of Interest Statement

The authors declare that the research was conducted in the absence of any commercial or financial relationships that could be construed as a potential conflict of interest.
